# Making sense of fragmentation and merging in lineage tracing experiments

**DOI:** 10.3389/fcell.2022.1054476

**Published:** 2022-12-14

**Authors:** Yiteng Dang, Steffen Rulands

**Affiliations:** ^1^ Max-Planck-Institute for the Physics of Complex Systems, Dresden, Germany; ^2^ Center for Systems Biology Dresden, Dresden, Germany; ^3^ Max-Planck-Institute for Molecular Cell Biology and Genetics, Dresden, Germany; ^4^ Arnold-Sommerfeld-Center for Theoretical Physics, Ludwig-Maximilians-Universität München, München, Germany

**Keywords:** lineage tracing, stem cells, development, wound healing, cancer, stochastic modelling, statistics

## Abstract

Lineage tracing experiments give dynamic information on the functional behaviour of dividing cells. These experiments therefore have become an important tool for studying stem and progenitor cell fate behavior *in vivo*. When cell proliferation is high or the frequency of induced clones cannot be precisely controlled, the merging and fragmentation of clones renders the retrospective interpretation of clonal fate data highly ambiguous, potentially leading to unguarded interpretations about lineage relationships and fate behaviour. Here, we discuss and generalize statistical strategies to detect, resolve and make use of clonal fragmentation and merging. We first explain how to detect the rates of clonal fragmentation and merging using simple statistical estimates. We then discuss ways to restore the clonal provenance of labelled cells algorithmically and statistically and elaborate on how the process of clonal fragmentation can indirectly inform about cell fate. We generalize and extend results from the context of their original publication.

## 1 Introduction

The development and maintenance of tissues relies on the tight regulation of cell migration, proliferation, differentiation and death. Cellular behaviour is typically linked to a progression through cell states in a lineage hierarchy, from multi-potent stem and progenitor cells to terminally differentiated cells. Research on the cellular programs that underlie these cell states has historically focused on molecular processes, such as the expression of genes in gene regulatory networks and epigenetic modifications of the DNA and chromatin. Recent advances in single-cell genomics have given rise to unprecedented possibilities in identifying these molecular states and associated molecular markers of cell states (Tam and Ho (2020); Tanay and Regev (2017); Stuart and Satija (2019)). Detailed molecular profiles do not, however, inform about the functional consequences of molecular cell states in terms of cell migration, proliferation, differentiation and death. By quantifying the enrichment of marker genes obtained from single-cell sequencing compared to lists obtained from perturbation experiments cellular function can be inferred statistically in a correlative manner (Ashburner et al. (2000)).

A more direct study of the functional behaviour of cells requires time-resolved measurements on the functional level. Despite recent advances in microscopy, live imaging remains highly challenging in most tissues. Lineage tracing experiments, where a subset of cells is genetically labelled, give dynamic information on a functional level *in vivo* (Blanpain and Simons (2013); Simons and Clevers (2011)). In these experiments, transgenic mouse models are used to label a subset of cells with genetic markers. These markers are bequeathed to all progeny of a labelled cell, termed a clone. Lineage tracing comes in many flavours: single and multi-color constructs, genomic barcoding, combined with genetic perturbations (Rulands and Simons (2016); Yum et al. (2021)). All of these methods have in common that the number of cells in a given clone, the clone size, reflects a history of cell division, differentiation or cell death events between the time point of initial labelling (induction) and the time point of analysis (Ruske et al. (2020)). While a given clone size is compatible with multiple histories between labelling and analysis, statistical ensembles of clones allow to rigorously infer mechanisms of cell fate behaviour and lineage relationships from clonal fate data. As an example, the average size of clones is related to the rate and mode of cell proliferation. For example, in homeostasis, the balance between clonal loss and growth leads to a linear increase of the average clone size (Klein and Simons (2011)). If this balance is tilted towards symmetric proliferation, such as is typical in early development or cancer, clone size distributions increase exponentially (Alcolea et al. (2014)). The probability distribution of clone sizes in addition reflects how cell fate is regulated, such as *via* extrinsic feedback or intrinsically. Methods from statistical physics and statistics have been successfully employed to unveil mechanisms of cell fate decisions in numerous tissues and contexts (Snippert et al. (2010); Doupé et al. (2010); Driessens et al. (2012); Shakiba et al. (2019); Scheele et al. (2017); Robertson et al. (2022)). Theoretical work has also highlighted generic features of clonal fate data (Klein et al. (2007); Yamaguchi et al. (2017); Rulands et al. (2018); Corominas-Murtra et al. (2020); Greulich et al. (2021)). Both in homeostasis and during tissue expansion clone size distributions converge over time to shapes that only partially depend on the details of cell fate regulation.

**FIGURE 1:**
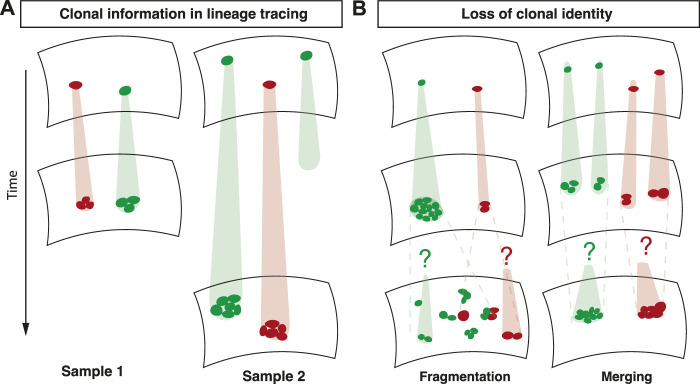
Overview of lineage tracing. **(A)** In lineage tracing experiments, a subset of cells is induced with an inheritable marker. The clonal relationship between an induced cell and its quantified progeny is used to unveil cell fate behavior. **(B)** Fragmentation and merging of clones renders the clonal identity of cells ambiguous.

The dynamic information encoded in clonal fate data relies on the strict clonal relationship between a group of cells at the time of analysis and a single induced cell at an earlier time point (Figure 1A). The loss of this association significantly complicates the interpretation of lineage tracing experiments. This association can be lost in two ways: First, at the time point of analysis, a group of cells might be associated with multiple induced cells (merging) (Figure 1B). In tissues, where clonal identity is usually defined by spatial proximity of groups of labelled cells, clonal merging is a consequence of the joining of multiple clones labelled with the same fluorescent marker. Second, multiple groups of cells at the time of analysis might be associated with a single induced cell (fragmentation) (Figure 1B). Clonal fragmentation is a consequence of large-scale tissue rearrangements, cell migration, or stochastic forces by cell divisions and cell loss (Ranft et al. (2010)). Cell intercalations, also termed T1 transitions, where cells rearrange and change neighbours, are also among the drivers of clonal fragmentation (Bocanegra-Moreno et al. (2022)). Clonal fragmentation therefore is particularly prominent in tissues with a high rate of cell proliferation, such as in development, regeneration or in tumours.

What can be learnt from lineage tracing data in these scenarios? A combination of theoretical and experimental work has shown that the ensuing size distributions tend to take a universal shape that is independent of the biological processes governing cell fate (Rulands et al. (2018)). This is because these processes lead to variability in clone sizes that dominates variability caused by other, cell-fate associated processes. Therefore, the merging and fragmentation of clones leads to the erasure of biological information contained in clone size distributions. In order to safeguard against drawing unfounded conclusions from clonal fate data it is therefore necessary to faithfully detect clonal fragmentation and merging in lineage tracing experiments and to restore clonality using statistical inference methods.

Here, we discuss statistical strategies that have recently been successfully employed to estimate rates of clonal fragmentation and merging and to restore the clonal provenance of fragmented cell clusters in a broad range of tissues. Where applicable we generalise these results beyond their original biological contexts. We argue that an estimation of the rates of clonal fragmentation and merging is an important step in the analysis of all lineage tracing experiments. In order for these methods to be applied by biologists who conduct these experiments we here focus on simplicity, intuition and applicability rather than quantitative exactness. We also discuss how the contiguity and the shape of clones provide information about morphogenetic behaviours such as oriented cell division and cell intercalation (Kaucka et al. (2016)).

## 2 Sensing clonal fragmentation and merging

A qualitative assessment of the prevalence of clonal fragmentation and merging can be obtained by inspecting the shape of the probability distribution of the sizes of labelled cell clusters. Merging and fragmentation of clones lead to stereotypical shapes - in particular the log-normal shape - that can be indicative of these processes (Rulands et al. (2018)). In the following, we will discuss strategies of how the rates of merging and fragmentation in a given experiment can be estimated quantitatively.

### 2.1 Estimating the induction frequency

A given number of labelled cell clusters can originate from a combination of multiple induction, merging and fragmentation events. Therefore, an independent estimate of the induction frequency is an important step in the analysis of lineage tracing experiments with an uncertain degree of clonality. In Lescroart et al. (2014) the authors studied the temporal commitment of Mesp1 expressing progenitors to heart morphogenesis. They labelled cells at different time points during early gastrulation (E6.5-E7.5), where most of the labelled cells divide symmetrically and the processes leading to the formation of different heart compartments involve large-scale cell rearrangements. These processes lead to the fragmentation of labelled clones between the time of labelling and the time of analysis at E12.5. To deal with fragmentation, a possible strategy could be to aim to label 1 cell per heart on average, which would allow calculating the fragmentation rate by simply calculating the average number of clusters of labelled cells per sample. However, since induction is generally stochastic, this would have implied a significant fraction of unlabeled hearts of roughly 30%. Therefore, Lescroart et al. aimed for an average number of induced cells that is larger than one. Since in this case a given number of labelled cell clusters can originate from any combination of multiple induction and fragmentation events, the retrospective clonal analysis required an independent estimate of the induction frequency.

In order to obtain such an estimate, the authors made use of the multi-color labelling strategy based on the *Confetti* construct used in this study. If induction events in different colors are statistically independent, then the number of different colors in a sample follows a binomial distribution. For a labelling strategy with a total of *n*_*C*_ different colors the fraction of organs that is labelled in *n* different colors, *C*_*n*_ then is

**FIGURE 2:**
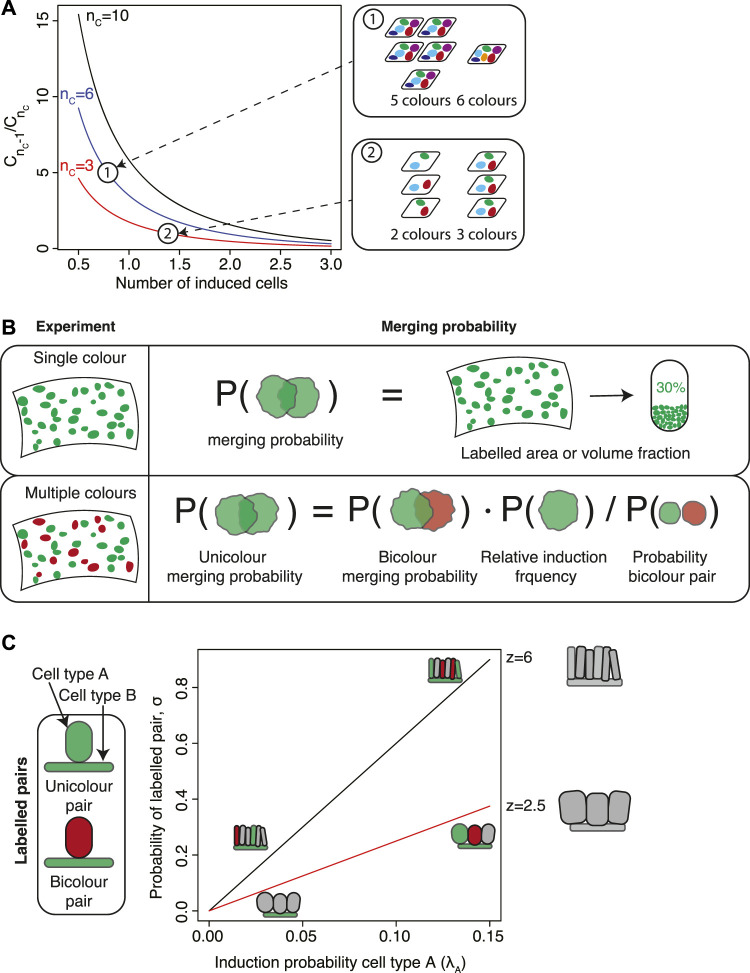
Estimating the induction frequency and merging probability. **(A)** In experiments involving multiple fluorescent markers, the induction frequency can be inferred by comparing the numbers of samples with different numbers of colors, denoted *C*_*n*_ for *n* colors. The ratio $C_{n_{C}-1}/C_{n_{C}}$ is most sensitive to the induction frequency at low values of the induction frequencies and in the case of many colors. **(B)** Calculating clonal merging rates from experiments. In unicolor experiments, the merging rate can be approximated as the area or volume fraction of the tissue that is labelled. In multicolor experiments, the unicolor merging probability is proportional to the bicolor merging probability times additional factors depending on the labelling frequencies of each individual color. **(C)** The probability that an induced cell of type B is in contact with an induced cell of type A depends the geometry of the tissue (coordination number) and the induction specificity. The insets show typical examples depicting different values of these parameters.

Cn=nCn1−JnJnC−n, (1)

where *J* is the probability that the tissue is unlabelled in a single color. If *m*_0_ denotes the average number of induced cells in a given tissue region then $J=e^{-m_{0}}$ (Table 1). If only labelled organs are recorded, then Eq. 1 needs to be divided by the probability that an organ is labelled at all, $1-J^{n_{C}}$ . While Eq. 1 cannot in general be solved for *m*_0_, we can solve for *m*_0_ in terms of the ratio *C*_*n*_/*C*_*n*+1_. Therefore, the ratio of the number of samples labelled in *n* colors, *C*_*n*_ and in *n* + 1 colors *C*_*n*+1_ can be used to infer the average number of labelled cells per sample, *m*_0_ (Figure 2A),

$$m_{0}=\log\left[1+\frac{n+1}{n_{C}-n}\frac{C_{n+1}}{C_{n}}\right].$$ (2)

**TABLE 1:** Notations used throughout this review.

*m*_0_	Parameter describing the average number of induced cells in a given experiment
*m*	Random variable describing the actual number of induced cells in a given sample
*k*	Random number describing the number of observed cell clusters in a given sample
*r*_*c*_	Probability that a given labelled cell has color *c*
*z*	Coordination number giving the number of neighbours a cell has
*n*_*c*_	Total number of colors expressed in a lineage tracing experiment
*f*	Average number of fragmentation events per clone between induction and analysis
*p*_*merge*_	Probability that a given cluster of labelled cells is polyclonal

In Lescroart et al. (2014) the authors applied this strategy by calculating the number of induced colors in all embryonic hearts analysed. In order to obtain a robust estimate of *m*_0_ this requires, however, a large number of animals. In larger tissues, for example in later stages of development or adulthood, an alternative strategy would be to define equally sized regions that are sufficiently separated to be statistically independent. Then one can quantify *C*_*n*_ and *C*_*n*+1_ across all of these regions. The average number of induced cells per region can then be calculated as above under the assumption that induction in all regions is statistically independent and with equal frequency.

### 2.2 Estimating the merging probability

The potential merging of induced clones disproportionally affects average clone sizes and the clone size distribution. For example, an estimate made from Monte Carlo simulations yields that a merging probability of 10% leads to a 20% increase in the average clone size. This is because larger clones have a higher probability of merging. Therefore, since the number and positions of induced clones are generally stochastic even for seemingly clonal experiments the merging probability should be controlled statistically. With an estimate of the induction frequency at hand the probability that a given cluster of labelled cells is polyclonal (merging probability) can, in principle, be estimated by comparing the number of induced cells with the number of observed cell clusters. If the number of observed cell clusters is significantly lower than the estimated number of induced cells then clonal merging is prevalent. Since this approach is based on a comparison between the time point of induction and the time point of analysis it implicitly relies on assumptions about the kinetic processes influencing the number of labelled cell clusters. Specifically, it neglects the possibility of clonal loss and fragmentation. In the following, we will discuss how the merging probability can be estimated independently of such assumptions.

#### 2.2.1 Multi-color experiments

For multi-color experiments the probability of clonal merging can be obtained elegantly by comparing mergers between cell clusters of different colors. As a first rough estimate, one could make use of two different colors, *c*_1_ and *c*_2_, that are expected to be induced with similar frequencies. Then, the merging probability of two clones with color *c*_1_ (unicolor mergers) is roughly equal to the probability that a cell cluster labelled in color *c*_1_ is in spatial contact with a cell cluster of color *c*_2_ (bicolor mergers). The latter is equal to the fraction of cell clusters of color *c*_1_ that are bicolor mergers, which can be easily be quantified from microscopy images.

If colors are not induced at similar frequencies, a more detailed analysis is necessary. Aragona et al. (2017) performed lineage tracing to study fate decisions of epidermal cells in the mouse tail during wound healing. They labelled interfollicular *epidermis* (IFE) stem cells using a *Confetti* labelling strategy, and observed that the induced IFE cells formed elongated stripes that extended towards the center of the wound. In order to distinguish monoclonal stripes from stripes originating from the merger of multiple stripes, they devised a statistical framework to calculate merging rates in multicolor clonal data. To this end, Aragona et al. calculated the correction that is to be made to the heuristic argument presented in the previous paragraph due to the fact that the fraction of bicolor mergers is in itself influenced by the presence of mergers. If *k*_*bicolor*_ is the number of observed clusters that are bicolor mergers between a color *c* and any other color and *k* is the total number of clusters, then the fraction of unicolor mergers is

$$G_{\mathrm{unicolor}}^{c}\approx \frac{k_{\mathrm{bicolor}}}{k}\frac{r_{c}}{1-\sum_{c}r_{c}^{2}}.$$ (3)

The nominator of the second term, *r*_*c*_, is the relative frequency of clones of color *c* which rescales the fraction *k*_*bicolor*_/*k* by the relative contribution of color *c*. The denominator corrects for the possibility of mergers when counting *k* (Figure 2B). This correction is particularly relevant if colors are not equally frequent. For example, in Aragona et al. (2017) the authors find prevalence of CFP, RFP, YFP clones at roughly 30% each and GFP at roughly 10%, which corresponds to a correction factor to the merging rate of roughly 0.72. For the labelling strategy used in Baggiolini et al. (2015); Kaucka et al. (2016), migratory neural crest cells induced at low recombination density express RFP at roughly 73%, YFP at 17%, CFP at 9% and GFP at less than 1%. This gives a correction factor of 0.43.

#### 2.2.2 Single-color experiments

Calculating the rate of clonal mergers in experiments with a single fluorescent color is much more challenging. In this case, the merging rate must be inferred from statistical models that are based on simplifying assumptions about clonal induction events and the spatial location of clones. Fortunately, in many cases the assumptions that clonal induction events are statistically independent and spatially uniform and that clonal expansion is spatially isotropic are approximately valid. They therefore serve as a starting point for statistical frameworks of clonal merging.

An intuitive argument already gives an estimate of the merging probability. For this argument, let us consider a tissue with a number of labelled clones. Let us pick out a random clone and put it in a random location in the tissue. What is the probability that this clone is put onto a location that is covered by another clone? If we neglect the spatial extension of the randomly picked clone then this probability is equal to the fraction of the tissue occupied by labelled cells (Figure 2B). Therefore, to quantify the degree of clonal merging an estimate can be obtained from a simple quantification of the area or volume fraction of labelled cells.

A similar argument was employed in Frede et al. (2016) who studied oesophageal tumour growth and observed a small number of clones that were much larger than the typical clone size. They reasoned that these clones might be a result of merging events and estimated the merging probability. Since the circumference of the tumour lesions they studied is effectively one-dimensional, the merging probability for a given clone size *s* is equal to the probability that the distance to the neighbouring clone is smaller than *s*. If *p* is the probability that a cell is induced this is equal to 1 − (1 − *p*)^*s*^. Taking into account that clone sizes are variable and are described by a distribution *P*(*s*), one then sums over contributions from different clone sizes,

$$p_{\mathrm{merge}}=1-\overset{\infty }{\underset{s=1}{\sum }}{\left(1-p\right)}^{s}P\left(s\right).$$ (4)

This argument, which was formulated for one spatial dimension (the circumference of the lesion) can be generalised to planar tissues (two spatial dimensions) and volumnar tissues (three spatial dimensions). Under the conditions of statistical independence and spatial uniformity of induction events and isotropy of clone expansion, the probability of clonal merging events can be estimated by noting that the strongest contribution to the merging probability comes from merging events with nearest neighbouring clones, while merging events between three or more clones are much less probable. Then, the probability of merging is equal to the probability that the distance between the centers of nearest neighbours is smaller than the sum of their radii. From this we obtain as a generalisation of Eq. 4,

$$p_{\mathrm{merge}}=\int_{0}^{\infty }\left(1-e^{-\rho V_{d}\left(r\right)}\right)C\left(r\right)\text{d}r.$$ (5)

where *ρ* = *m*_0_/*V* is the density of clones in the tissue, $V_{d}\left(r\right)=\pi^{\frac{d}{2}}r^{d}\Gamma \left(\frac{d}{2}+1\right)$ is the volume of a sphere of radius *r* in *d* spatial dimensions, where Γ is the Gamma function and *C*(*r*) is the probability that the sum of two radii is equal to *r* which is derived from the clone size distribution. This means that, similar to Frede et al. (2016), the merging probability can in general be obtained from an estimate of the induction frequency, *m*_0_, and the clone size distribution.

### 2.3 Application to multipotency

An important application of lineage tracing experiments is to establish lineage relationships between different cell types. The idea is that if an induced cell type gives rise to clones containing another type of cells then the latter is lineage related to the former. However, if induction is not entirely specific such that both types of cells are induced, then the chance of merging of clones might lead to unguarded conclusions about multipotency. Therefore, in order to establish the hypothesis of multipotency one needs to devise a statistical test quantifying the significance of an enrichment of clones containing both cell types compared to clonal merging by chance.

Wuidart et al. (2016) devised a statistical framework to test for multipotency which they applied to mammary gland and prostate tissues, two ductal epithelial tissues where basal cells and luminal cells are in direct contact with one another (Figure 2C). If induction events are statistically independent the probability that a given pair of basal and luminal cells are chance mergers is binomial. This constitutes the null hypothesis. Therefore, a binomial test can be used to establish the statistical significance of the alternative hypothesis of multipotency. If k* is the number of observed pairs of basal and luminal cells of the same color, the *p*-value is

$$p=\overset{N}{\underset{k=k^{*}}{\sum }}\left(\begin{array}{c} N\\ k \end{array}\right)\;\nu^{k}{\left(1-\nu \right)}^{N-k}.$$ (6)

The value of the sample size *N* and the parameter *ν* depend on the statistical model used to describe the null hypothesis.

Based on this idea, Wuidart et al. derived two orthogonal statistical tests. In the first test, they considered only pairs of labelled basal and luminal cells. The null hypothesis then is that among these pairs the ones that are of the same color arise by chance labelling events. In this case, *ν* is the probability that a given pair of labelled basal and luminal cells is of the same color. This probability is equal to

$$\nu =\underset{C}{\sum }r_{C}^{B}r_{C}^{L},$$ (7)

where $r_{C}^{A}$ and $r_{C}^{B}$ the relative probabilities of labelling luminal and basal cells in color *c* respectively. As an example, in an experiment using the *Confetti* construct on two cell types A and B, the majority colors RFP, YFP and CFP are approximately induced at the same probability $r_{C}^{A}=1/3$ and $r_{C}^{B}=1/3$ for all colors and consequently *ν* = 1/3. Then, if 50 out of 100 pairs are unicolor, *k* = 50 and *N* = 100 such that the *p*-value, Eq. 6, is *p* = 0.0004.

This example demonstrates that although this test is insensitive to assumptions about the tissue architecture and overall labelling frequencies, it requires a large number of unicolor pairs in order to establish statistical significance. If induction frequencies are low these pairs are rare such that a large number of tissues potentially needs to be analysed.

For a broader statistical test, Wuidart et al. used the fraction of basal cells in contact with a luminal cell of any color. Under the null hypothesis of chance merging, this probability depends on the geometry of the tissue, which is encoded in the coordination number *z* giving the number of luminal cells in contact with one basal cell. As the coordination number may differ between individual basal cells, we define the distribution of coordination numbers, F(z) , which can be directly obtained experimentally from microscopy images. The probability that an induced basal cell is paired to a labelled luminal cell then takes the rather lengthy form

$$\nu =\left(\underset{C}{\sum }\lambda_{C}\right)\overset{\infty }{\underset{z=1}{\sum }}\left[z{\left(1-\underset{C^{\prime }}{\sum }\lambda_{C^{\prime }}\right)}^{z-1}F\left(z\right)\right],$$ (8)

which now depends on the luminal induction probability, *λ*_*C*_, which should be determined independently. If variations in coordination number are small we can approximate the pairing probability as

$$\nu \approx \bar{z}\underset{C}{\sum }\lambda_{C},$$ (9)

where $\bar{z}$ denotes the average coordination number. Therefore, in this case the pairing probability depends only on the geometry of the tissue and the specificity of the genetic labelling construct. Substituting this into Eq. 6 gives another binomial test for mulipotency where k* now is the number of labelled basal cells in contact with a labelled luminal cell and *N* is the total number of labelled basal cells. A similar approach was also followed in Lilja et al. (2018) in order to quantify the degree of bipotency in the mammary gland during different time points in development.

These tests are not restricted to tissues containing basal and luminal cells, but can be applied and generalised to any tissue where cell layers are in direct contact with each other. Since both tests rely on complementary aspects of the clonal data it is advisable to strengthen one’s conclusion about multipotency by performing both of them. Should these tests indicate unipotency, then a direct comparison between the predicted values of Eqs 7–9 with experimental data could further strengthen this conclusion. In the case that the analysis supports multipotency, these comparisons can allow inferring the rate of differentiation between both cell types.

#### 2.3.1 Clonal merging in barcoding experiments

In recent years, fluorescent-marker-based assays have been supplemented with labelling strategies using nucleic-acid-based barcoding (Kebschull and Zador (2018); Wagner and Klein (2020)). Due to the large number of induced cells, these approaches require the creation of a barcode library with sufficient diversity to induce individual cells with unique barcodes, as well as a sequencing-based method to read out the barcodes. *In vivo* barcoding methods generally fall into one of two classes: 1) methods based on recombinase enzymes, which flip or excise DNA sequences to generate sequence diversity and 2) CRISPR-based methods where an endonuclease enzyme such as Cas9 introduces insertions or deletions into a genomic region of the DNA. Readout of the barcodes can be performed by PCR amplification followed by bulk sequencing, through single-cell RNA sequencing, through fluorescence *in situ* hybridization (FISH) methods or even through spatial transcriptomics (Rao et al. (2021)).

While typically a large number of different barcodes is generated, not all of them are induced with the same probability. This gives rise to the chance induction of several cells with the same barcode and hence clonal merging. The non-spatial methods discussed in Sections 2.1–2.3 can be applied to barcoding experiments, where different barcodes take the role of fluorescent colors. Alternative approaches rely on modelling the molecular processes leading to the generation of barcodes in order to estimate the induction frequency of individual barcodes (Marcou et al. (2018)).

### 2.4 Estimating the fragmentation rate

Estimating the rate of clonal fragmentation is not only important to understand the degree of clonality of a given experiment, but can also be informative for understanding the underlying processes that cause fragmentation (*cf.* Section 3.3). In order to estimate the rate of fragmentation, Lescroart et al. (2014) derived the probability distribution *F*(*k*) of the number of fragments *k* in a given sample. Since this distribution depends both on the average number of fragmentation events between labelling and analysis, *f*, and the number of induced cells, *m*, it in principle allows inferring both simultaneously from clonal fate data. To this end, they defined the joint probability of inducing *m* cells and observing *k* fragments, *J* (*k*, *m*). This joint probability is equal to the conditional probability of observing *k* fragments given *m* induced cells multiplied with the marginal probability of inducing *m* cells. Since individual induction and fragmentation events are considered to be statistically independent, both follow Poisson distributions, such that

$$J\left(k,m\right)=\frac{{\left(mf\right)}^{n-m}}{\left(k-m\right)!}\frac{m_{0}^{m}}{m!}e^{-mf-m_{0}}.$$ (10)

Because completely unlabelled organs are often not quantified, this needs to be divided by the probability that a tissue is unlabelled, *J* (0,0). Taken together, the number of fragments then follows

$$F\left(k\right)=\frac{\sum_{m=1}^{k}J\left(m,k\right)}{J\left(0,0\right)}.$$ (11)

With this, the fragmentation rate *f* and the induction frequency *m*_0_ can be estimated from the data, for example by maximum likelihood estimation.

**FIGURE 3:**
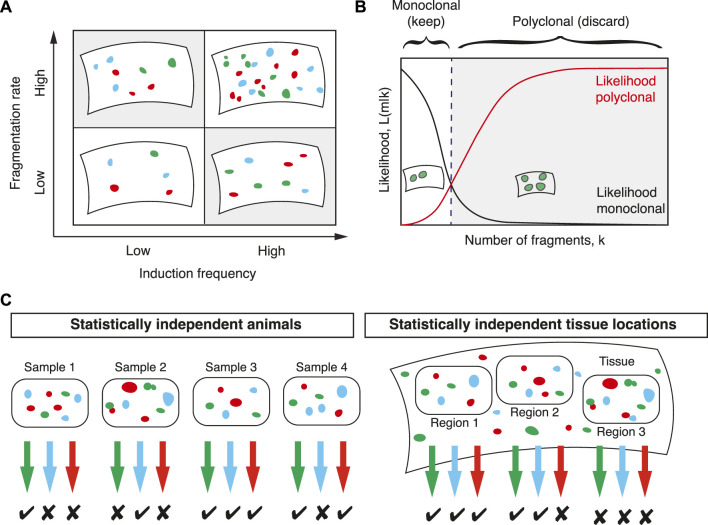
Computing clonal fragmentation rates. **(A)** The number of labelled cell clusters depends in similar ways on the induction frequency and the fragmentation rate (shaded areas). Since both are *a priori* unknown their simultaneous inference is challenging. **(B)** To determine whether a sample is monoclonal, a statistical framework based on comparing the likelihood that a sample is monoclonal (black line) with the likelihood that it is polyclonal (red line) defines a threshold (dashed line) in the number of fragments that separates samples that are considered monoclonal or polyclonal. **(C)** For each sample colors with more fragments than the threshold value defined in **(B)** are discarded. Samples can be tissues from different animals or large, but spatially separated and statistically independent tissue regions from the same animal. In this example, samples with two or fewer fragments are considered monoclonal.

Because two parameters with similar effect on the distribution *F*(*k*) need to be estimated simultaneously (Figure 3A), it is expected that with the sizes of typical lineage tracing experiments the uncertainties associated with these estimates are large. Therefore, in the case of multi-color labelling, the induction frequency should be estimated independently as in Section 2.1. Then, in a second step, the fragmentation rate *f* can be inferred more robustly.

## 3 Interpreting nonclonal experiments

Given that merging or fragmentation of clones confound a lineage tracing experiment, how can information about cell fate behaviour still be inferred? In the following, we will discuss three approaches that solve this problem in different ways: by discarding nonclonal experimental samples, by restoring the clonal origin of labelled cells and by taking clonal fragmentation as an opportunity to learn about the mechanical effects of cell fate decisions.

### 3.1 Filtering for monoclonal samples

In the first approach, samples with fragments deriving from multiple induction events are filtered out and the downstream analysis is restricted to monoclonal samples (Figure 3B). This approach was followed in Lescroart et al. who devised a statistical framework that allows, with known uncertainty, to filter for monoclonal samples. The decision of whether a sample is monoclonal is quantified by the ratio of two probabilities: the probability that a given number of fragments are polyclonal and the probability that a given number of fragments are monoclonal. Following Bayes’ theorem, this ratio is equal to the ratio of two likelihood functions: if $L\;\left(k|m\right)$ should be $L\left(k|m\right)$ is the probability that an observed number of *k* fragments arises from *m* labelled cells then this ratio is

$$\Lambda =\frac{\sum_{m=2}^{\infty }L\left(m|k\right)}{L\left(m=1|k\right)}.$$ (12)

By defining a threshold value of Λ this defines a number of fragments in a given color below which a given color in a given sample is considered monoclonal (Figure 3B). If Λ ≥ 1 a given sample is more likely to be polyclonal than monoclonal and these samples were discarded in Lescroart et al. (2014). To achieve a given higher significance level (a stricter criterion for monoclonality) a smaller number than one can be chosen as a threshold at the cost of a larger number of discarded data points. With this threshold defined, the experimental data can then be filtered for samples containing fewer fragments than this threshold (Figure 3C). All downstream analysis then only considers samples that are monoclonal with defined uncertainty. Lescroart et al. (2014) used hearts from different animals and different fluorescent colours to define such samples. An alternative approach is, as mentioned in Section 2.1, to define regions in the tissue that are much larger than the typical expected size of dispersed clones and sufficiently distant to be considered statistically independent (Figure 3C).

### 3.2 Restoring clonality

If the rates of merging and fragmentation are small, clonality can potentially be restored statistically by making use of the fact that labelled cells belonging to the same clone have the tendency to be located closer to each other compared to cells originating from different induction events. As a first approach one might therefore employ standard clustering techniques, such as hierarchical clustering or k-means clustering on the spatial coordinates of labelled cells. These approaches, however, have shortcomings in the context of lineage tracing experiments: First, they often lack unambiguous ways of determining the total number of induced clones, which then becomes an independent parameter that needs to be determined using different tools (*cf.* Section 2.1). Secondly, they lack ways for quantifying levels of uncertainty associated with the assignment of cells to clones. Importantly, these uncertainties need to be propagated to downstream analyses, such as the calculation of average clone sizes. Transparency about uncertainties also allows one to be tolerant with respect to mis-assignments in clonal identity. Thirdly, standard clustering algorithms usually do not allow to take into account additional information experimentalists might have on a specific biological context, such as on the rates of cell migration.

Than-Trong et al. (2020) studied cell fate behvaiour of neural stem cells in the Zebrafish brain. Because labelled clones dispersed over time, they devised a statistical framework that overcomes these limitations by combining a biophysical model of clone dispersion with Bayesian inference. The key idea is to calculate the probability that a given partition of labelled cells into clones is the true clonal partition (posterior). According to Bayes’ theorem, this probability is proportional to the probability that a given clonal partition arises from statistically independent induction events (termed likelihood) multiplied with the *a priori* probability that a given partition arises in the first place (termed prior). The likelihood was modelled as a stochastic process of clonal induction and dispersion while the prior contains information about clonal induction frequencies or clone dispersion estimated independently from the data. Then, the partition of cells into clones that maximized the posterior, and its uncertainty, was approximately obtained by successively coarse graining cells in larger and larger clones.

In a more simplistic way, experimentalists quantifying clonal fate data could also decide on the clonality of a group of fragments based on the distance between these fragments. In order to specify a distance w* below which a pair of fragments can be considered clonal we consider a pair of fragments and assume that these fragments are nearest neighbors. Then, if we aim for a given fraction *α* of polyclonal cell clusters at a distance shorter than w* (significance level), we get as a condition

$$\int_{0}^{w^{*}}P\left(m=2|w\right)P\left(w\right)\text{d}w=\alpha ,$$ (13)

where *P* (*m* = 2|*w*) is the probability that two fragments at a given distance *w* arise from two separate induction events and *P*(*w*) is the probability to find any two fragments at distance *w*. According to Bayes’ theorem this is equal to $\int_{0}^{w^{*}}P\left(w|m=2\right)P\left(m=2\right)\text{d}w$ , where *P* (*w*|*m* = 2) is the probability that a pair of monoclonal fragments have a distance *w*. If clone induction events are statistically independent then $P\left(w|m=2\right)=e^{-\rho \pi^{d/2}w^{d}/\Gamma \left(d/2+1\right)}$ (Diggle (2013)), where *ρ* = *m*_0_/*V* is the density of clones, *V* is the area or volumes analysed, and Γ is the Gamma function. *P* (*m* = 2) is the *a priori* probability that two clusters of labelled cells at any distance arise from two separate induction events. If the fragmentation rate is known this is equal to the probability that an induced clone has fragmented, *e* ^*f*^. Taken together, we can solve for w* and obtain

$$w^{*}={\left[\frac{\Gamma \left(d/2+1\right)\left(f-1\right)V}{\pi^{d/2}k\ln\left(1-\alpha e^{-f}\right)}\right]}^{1/d}.$$ (14)

As expected, the distance threshold w* decreases with the number of fragmentation events between induction and analysis, *f*, and with the overall number of cell clusters, *k*. w* is the distance below which two labelled cell clusters are clonal with significance level *α*. A reasonable choice could be *α* = 0.5 such that at distances shorter than w* two fragments are more likely clonal than polyclonal.

### 3.3 Learning from clonal fragmentation about cell fate

Instead of treating clonal fragmentation as a liability for the analysis of clonal fate data, in recent years several groups have taken an orthogonal perspective. They noted that the dispersion of clones after labelling reflects a history of mechanical forces acting on the cells in the clone and that an analysis of clonal fragmentation can give insights into these processes. Watson et al. (2020) used a zebrafish line that fluorescently labels osteoblasts and then used CRISPR to induce mutations of Plod2 and Bmp1a in a subset of cells. The ensuing clone size distribution takes the universal shape as predicted to arise from fragmentation and merger events (Rulands et al. (2018)). Therefore, clone size distributions were not informative about the phenotype. However, by using statistical measures for the spatial distribution of labelled cells they could identify systematic differences between wildtype and mutant samples. One of these measures is termed Moran’s I which is a statistical measure of spatial correlations. The authors further used multivariate statistical tests to compare the spatial fluorescence signal between conditions and performed Monte Carlo simulations. This work exemplifies that while clone size distributions may converge to universal shapes that are independent of the biological condition, quantities capturing the spatial statistics of labelled cells may carry cell fate specific information.

Further works associated clonal fragmentation with mechanical processes in tissues. Ramanathan et al. (2019) used models from statistical mechanics to show that clone dispersion is inversely proportional to cell size, such that smaller cells show higher clonal dispersion. In these models, termed vertex models, cells are considered as polygons with vertices and edges representing the boundaries between cells Farhadifar et al. (2007). The location of vertices and edges then follows equations of motion determined by different forces acting on vertices. Numerical results obtained from these models were also compared to experimental findings from the *Drosophila* wing disk of mutant embryos with smaller cells.

Bocanegra-Moreno et al. (2022) took a more direct approach of inferring mechanical and material properties from clonal fragmentation. They performed lineage tracing using the MADM system (Zong et al. (2005)) in the mouse neural tube. Based on these experiments they used information on the fragmentation of clones to infer mechanical and material properties of tissues, and to learn about cellular processes that control fragmentation. Specifically, in order to compare patterns of clone fragmentation with theoretical predictions, they implemented a vertex model parametrised by normalised tension and normalised contractility, and applied noise to the normalised tension. In their simulations they labelled individual cells and tracked how the progeny of these cells dispersed in the tissue. While clonal fragmentation alone was not sufficient to determine the position of the tissue in parameter space, the combination of fragmentation, the rate of T1 transitions - where cells rearrange and change neighbours - and geometric parameters describing cell shapes allowed them to identify tissue and material properties of the neural tube. They also found *in vivo* and *in silico* that the fragmentation rate increases with the rate of cell divisions, while cell differentiation leads to a reduction in clone fragmentation.

Kaucka et al. (2016) combined lineage tracing, computer simulations, live imaging and mouse mutants to study clonal dynamics and cell movements of the cranial neural crest cells that give rise to the ectomesencyhme. They found that clones originating from a single neural crest cell form clonal envelopes, well-defined clusters of labelled cells that remain stable over time. However, these clusters overlap with one another, such that there is considerable mixing between cells from different clones. Computational modelling suggested that pushing of cells arising from cell division rather than cell migration is responsible for generating this clonal mixing. This is confirmed through live imaging, which revealed crowd motion of these clones with limited individual cell migration. Furthermore, they also find evidence of oriented cell divisions, which are consistent with a computational model that includes a signalling gradient, which in turn would explain the elongated shapes of clones they observed. Taken together, these works show that merging and fragmentation processes in themselves carry valuable information about the mechanical processes that underlie tissue morphogenesis.

## 4 Discussion

The intepretation of genetic tracing experiments relies on the integrity of the clonal relationship between induced cells and their progeny at the time point of analysis. Large-scale cell rearrangements or stochastic forces in the tissue lead to the fragmentation and merging of clones, which renders the retrospective analysis of such experiments highly ambiguous. This is on the one hand because the resulting size distributions obtain universal shapes that are largely independent of cell fate behaviour. On the other hand, since the induction frequency and the merging and fragmentation rates are *a priori* unknown, restoring the clonal origin of induced cells is statistically challenging.

If such non-clonality is undetected it might lead to unguarded claims about cell fate behavior. In this review, we discussed statistical strategies to detect non-clonality in genetic tracing experiments. Genetic constructs where clones can be induced in multiple colors provide simple and elegant routes to estimating the induction frequency by comparing the fractions of samples induced in different numbers of colours. They also allow for estimating the merging rate by counting the number of mergers between cell clusters of different colours. For experiments using only a single color, estimates of merging and fragmentation rates are feasible using stochastic models of clonal induction and *a priori* knowledge about the clone size distribution. Given that estimating the merging and fragmentation rates is feasible with no additional experimental effort, we suggest that such estimates should be conducted as a safety check in the analysis of clonal fate data. With the advent of single-cell genomics and deep sequencing, strategies that label clones with fluorescent colors have been complemented with nucleic-acid based barcoding. Since these barcodes are induced at different, not neccessarily clonal frequencies, multiple cells can be labelled with the same barcode. From a statistical perspective this again leads to the effective merging of clones. By treating barcodes as different colours the statistical tools presented here to detect merging in multi-color experiments can be used to estimate the degree of clonality in barcoding experiments.

If clonal merging or fragmentation has been detected, several strategies exist for interpreting nonclonal data. A first strategy relies on filtering for monoclonal fragments. This approach is applicable even if the fragmentation rate is high, but it comes with the downside that a potentially large fraction of the data is not used for further analysis. If fragmentation and merging are infrequent, such that the number of fragmentation events is much smaller than the number of cell divisions in a given time interval, the clonal provenance of labelled cell clusters can be faithfully restored by statistical inference or through setting simple thresholds in the distances between labelled cells. While these approaches cannot restore clonal information that is ultimately lost due to strong dispersion, they do come with well-defined statistical uncertainties in the assignment of cells to clones. As long as these uncertainties associated with the assignment of cells to clones are transparently propagated, occasional mis-assignments of the clonal origin of labelled cells do not compromise the interpretability of downstream results.

While clonal fragmentation and merging renders the *sizes* of clones ambiguous, valuable information about cell fate might be contained in other statistical quantities of labelled cell clusters. First, the rates of merging and fragmentation and their respective changes over time are associated with the rates of cell proliferation and cell rearrangements in the tissue. Further, the *shapes* of clones reflect anisotropic forces which are relevant for understanding morphogenesis. Such forces may arise through oriented cell divisions (Economou et al. (2013)), but also through collective motion leading to morphogenetic flows. In order to quantitatively interpret these features and to deduce mechanisms of tissue morphogenesis, models describing the collective effects of mechanical forces in tissues need to integrate clonal dynamics. Here, a promising line of research will be the integration of the shapes and boundary roughness of labelled cell clusters into quantitative frameworks of tissue mechanics.
